# Observation of a Higher‐Order End Topological Insulator in a Real Projective Lattice

**DOI:** 10.1002/advs.202303222

**Published:** 2024-01-12

**Authors:** Ce Shang, Shuo Liu, Caigui Jiang, Ruiwen Shao, Xiaoning Zang, Ching Hua Lee, Ronny Thomale, Aurélien Manchon, Tie Jun Cui, Udo Schwingenschlögl

**Affiliations:** ^1^ King Abdullah University of Science and Technology (KAUST) Physical Science and Engineering Division (PSE) Thuwal 23955‐6900 Saudi Arabia; ^2^ State Key Laboratory of Millimeter Waves Southeast University Nanjing 210096 China; ^3^ Institute of Artificial Intelligence and Robotics Xi'an Jiaotong University Xi'an 710049 China; ^4^ Department of Physics National University of Singapore Singapore 117551 Republic of Singapore; ^5^ Joint School of National University of Singapore and Tianjin University International Campus of Tianjin University Fuzhou 350207 China; ^6^ Institut für Theoretische Physik und Astrophysik Universität Würzburg 97074 Würzburg Germany; ^7^ CINaM Aix‐Marseille University CNRS Marseille France

**Keywords:** topological insulator, higher‐order, real projective lattice, bulk‐end correspondence, topolectric circuit

## Abstract

The modern theory of quantized polarization has recently extended from 1D dipole moment to multipole moment, leading to the development from conventional topological insulators (TIs) to higher‐order TIs, i.e., from the bulk polarization as primary topological index, to the fractional corner charge as secondary topological index. The authors here extend this development by theoretically discovering a higher‐order end TI (HOETI) in a real projective lattice and experimentally verifying the prediction using topolectric circuits. A HOETI realizes a dipole‐symmetry‐protected phase in a higher‐dimensional space (conventionally in one dimension), which manifests as 0D topologically protected end states and a fractional end charge. The discovered bulk‐end correspondence reveals that the fractional end charge, which is proportional to the bulk topological invariant, can serve as a generic bulk probe of higher‐order topology. The authors identify the HOETI experimentally by the presence of localized end states and a fractional end charge. The results demonstrate the existence of fractional charges in non‐Euclidean manifolds and open new avenues for understanding the interplay between topological obstructions in real and momentum space.

## Graphical abstract

A higher‐order end topological insulator in a real projective lattice is predicted and verified using topolectric circuits. The dipole‐symmetry‐protected phase manifests as 0D topologically protected end states and a fractional end charge. The discovered bulk‐end correspondence reveals that the fractional end charge, which is proportional to the bulk topological invariant, can serve as a generic bulk probe of higher‐order topology.

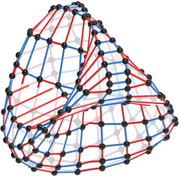

## 1 Introduction

A fundamental model of a topological phase^[^ ^1^, ^2^, ^3^, ^4^, ^5^, ^6^, ^7^, ^8^, ^9^, ^10^, ^11^, ^12^, ^13^ ^]^ is the 1D Su‐Schrieffer‐Heeger (SSH) model, characterized by a quantized dipole moment. Due to the presence of inversion symmetry,^[^ ^14^ ^]^ the bulk polarization (primary topological index) is 1/2 (unit: elementary charge *e*), resulting in a dipole‐symmetry‐protected phase with charges of ±1/2 at the ends of the 1D chain.^[^ ^15^ ^]^ Recently, the concept of dipole moment as bulk polarization was generalized to multipole moment, such as quadrupole and octupole moment, leading to the discovery of higher‐order topological insulators (HOTIs) characterized by a fractional corner charge (secondary topological index).^[^ ^5^, ^6^ ^]^ However, the predictive power of the fractional corner charge is limited in higher‐order topological crystalline insulators,^[^ ^16^ ^]^ where boundaries with higher co‐dimension do not always have in‐gap spectral features. For instance, the in‐gap edge states in the 2D SSH model do not carry the complete information of the bulk and the corner states cannot be isolated due to coincidental degeneracy with the bulk states.^[^ ^17^, ^18^ ^]^

Here, we put forward a higher‐order end TI (HOETI) in a real projective lattice (RPL) with (i) dipole‐symmetry protection, (ii) 0D end states, and (i) a fractional end charge of 1/2 (secondary topological index). We extend the first‐order bulk‐end correspondence, which refers to end states with co‐dimension one (as in the 1D SSH model), to higher order, i.e., end states with co‐dimension higher than one. The fractional end charge originates from the symmetry‐protected bulk charge, which can thus serve as a generic bulk probe of higher‐order topology, providing a means for characterizing crystalline insulators.

## 2 Results

**Figure 1:**
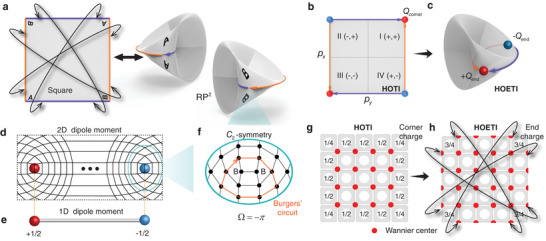
RP^2^ and HOETI. a) RP^2^ and its quotient space with the boundaries indicated by colors (orange and purple) and the corners indicated by letters (*A* and *B*). b) HOTI with edge polarizations (*p*_*x*_ and *p*_*y*_) and fractional corner charges (*Q*_corner_). c) HOETI with fractional end charges (±*Q*_end_). d) 2D dipole moment with fractional charges at disclinations. e) 1D dipole moment with fractional end charges of ±1/2. f) Zoomed view of the disclination with Frank angle Ω = −π. g) Filling anomaly of a HOTI with the Wannier centers contributing to both the fractional edge and corner charges. h) Filling anomaly of a HOETI with the Wannier centers contributing only to the fractional end charges, as the edges are glued together. Further details of Figures 1a,b, and d are available in Figures S1, S2, and S3 (Supporting Information), respectively.

We introduce the RPL as a quadrangular tiling of the real projective plane (RP^2^), which is a non‐Euclidean and non‐orientable manifold without boundaries. By Whitney's embedding theorem,^[^ ^19^, ^20^ ^]^ one cannot embed the RP^2^ in a 3D space without it intersecting itself, which inhibits physical realization. Instead of realizing the complicated manifold in its entirety, we can equivalently study its quotient space (the unfolded manifold equipped with gluing rules), i.e., a unit square ([0, 1] × [0, 1]) with opposite sides connected with a half‐twist, (0, *y*) ∼ (1, 1 − *y*) for 0 ⩽ *y* ⩽ 1 and (*x*, 0) ∼ (1 − *x*, 1) for 0 ⩽ *x* ⩽ 1 (twisted boundary conditions^[^ ^21^, ^22^, ^23^ ^]^). Diagonally opposite corners of the square are connected, forming two singularities in the RP^2^ (**Figure** **1**a). Accordingly, we can realize the RPL as a square lattice with real projective boundary conditions (twisted boundary conditions applied to both the *x* and *y*‐directions with one connection of the corners removed to ensure the homogeneousness of the geometry).

A quantized multipole insulator, as a pioneering example of HOTIs with in‐gap corner states, is difficult to be realized in a material due to its non‐commutative mirror symmetries.^[^ ^5^, ^6^ ^]^ HOTIs without quantized multipole moments (mostly higher‐order topological crystalline insulators) but with corner states embedded into the bulk spectrum have been proposed.^[^ ^18^ ^]^ In the schematic of such a HOTI^[^ ^24^, ^25^ ^]^ in Figure 1b, *p*_*x*_ and *p*_*y*_ are the edge polarizations and *Q*_corner_ denotes the corner charge. *Q*_corner_ can be calculated by integrating the charge density over the adjacent quadrant of the lattice. In quadrant III, for example, $Q_{{\text{corner}}^{\left(-x,-y\right)}}+Q_{{\text{edge}}^{-x}}+Q_{{\text{edge}}^{-y}}=\sum_{r_{x}=1}^{n_{x}/2}\sum_{r_{y}=1}^{n_{y}/2}\rho_{r}$, where ρ_*r*_ is the charge density, *r* = (*r*_*x*_, *r*_*y*_) is the lattice index with *r*_*x*_ ⩽ *n*_*x*_ and *r*_*y*_ ⩽ *n*_*y*_, ±*x* and ±*y* label the edges, and (±*x*, ±*y*) labels the quadrants of the square lattice. In the case of a HOETI, the in‐gap edge states of the HOTI are annihilated by the real projective boundary conditions and in‐gap end states are created by coupling the corners. In the schematic of a HOETI in Figure 1c, the edge polarizations vanish, which leaves the end charge decoupled from the bulk. The end charge is given by

(1) $$Q_{{\text{end}}^{\pm }}=Q_{{\text{corner}}^{\left(+x,\pm y\right)}}+Q_{{\text{corner}}^{\left(-x,\mp y\right)}}$$

where + denotes the *A*–*A* and − denotes the *B*–*B* end. *Q*_end_ can be calculated by integrating the charge density over diagonally opposite quadrants of the lattice. According to Figure 1d, there exists always an inversion‐symmetric pair of 0D ends (*A*–*A* and *B*–*B* with Frank angle Ω = −π, see Figure 1f and the Methods section for details). The 2D dipole moment of a HOETI is an analog of the 1D dipole moment shown in Figure 1e (e.g., dipole moment in the inversion‐symmetric 1D SSH model). The filling anomaly (mismatch between the number of electrons in the occupied bands and the number of electrons required for charge neutrality) of a HOTI (Figure 1g), which results from the fact that the Wannier centers are located at the corners of the unit cell, leads to a nontrivial phase with fractional corner charges of 1/4 and fractional edge charges of 1/2. In contrast, the filling anomaly of a HOETI (Figure 1h) leads to only fractional corner charges of 3/4, which are combined to give a fractional end charge of 1/2(=6/4mod1).

**Figure 2:**
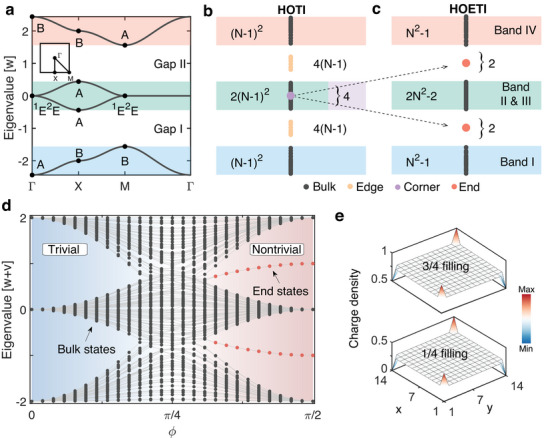
Topological properties. a) Tight‐binding band structure (*w* = 1, *v* = 0.22). The bands are labeled by the little group representations A, B, and ^1^E^2^E. b,c) Considering a lattice of *N* × *N* unit cells, the number of eigenstates below and above the energy gaps is distinct between a HOTI and a HOETI. The four degenerate partially occupied corner states of a HOTI are projected to become two pairs of end states of a HOETI. d) Eigenvalue spectrum of a HOETI as a function of ϕ. e) Spatial distributions of the charge density in a HOETI (7 × 7 unit cells) at 1/4 and 3/4 filling. The eigenvalue spectra, fillings, and spatial distributions of the charge density are compared in Figure S4 (Supporting Information) for the trivial and nontrivial phases.

We consider a *C*_4_‐symmetric tight‐binding model with four sites per unit cell, in which each site is strongly coupled to its nearest neighbors in adjacent unit cells (coupling *w*) and weakly coupled to its nearest neighbors within the same unit cell (coupling *v*). The obtained band structures are shown in **Figure** **2**a and can be characterized by the fractional corner charge

(2) $$Q_{\mathrm{corner}}=\frac{1}{4}\left(\left[X_{1}^{\left(2\right)}\right]+2\left[M_{1}^{\left(4\right)}\right]+3\left[M_{2}^{\left(4\right)}\right]\right)\;\mathrm{mod}\;1$$

deduced from the symmetry indicators,^[^ ^12^, ^26^, ^27^ ^]^ where $\left[\Pi_{p}^{\left(n\right)}\right]\equiv \#\Pi_{p}^{\left(n\right)}-\#\Gamma_{p}^{\left(n\right)}$ and $\#\Pi_{p}^{\left(n\right)}$ is the number of occupied bands at the high‐symmetry point Π (= X, M) with the *C*_*n*_ rotation eigenvalues *e* ^2πi(*p* − 1)/*n*^ (*p* = 1, …, *n*). The bulk‐corner correspondence yields *Q*_corner_ = 0 for the trivial phase and *Q*_corner_ = 1/4 for a HOTI. Since the middle two bands in Figure 2a touch each other at 1/2 filling, *Q*_corner_ obtained for the first occupied band cannot identify the specific arrangement of the corner states at zero energy.^[^ ^28^ ^]^ By imposing the real projective boundary conditions, the four degenerate partially occupied corner states with representation A⊕B⊕^1^E^2^E^[^ ^21^, ^22^, ^23^ ^]^ in Figure 2b are projected to become two pairs of end states with representations A⊕B and ^1^E^2^E, which are partially occupied at 1/4 and 3/4 filling (Figure 2c). To capture the previously identified topological end states, we extend the formulation of topological defect response (see the Methods section for details) as

(3) $$Q_{\mathrm{end}}=\frac{1}{2}\left(-\left[X_{1}^{\left(2\right)}\right]+2\left[M_{1}^{\left(4\right)}\right]+3\left[M_{2}^{\left(4\right)}\right]\right)\;\mathrm{mod}\;1$$

which manifests the bulk‐end correspondence and yields *Q*_end_ = 0 for the trivial phase and *Q*_end_ = 1/2 for a HOETI, making it possible to identify the bulk topology.

In the RPL, the discrete translation symmetry is broken and the topological phase can be analyzed by exact diagonalization to compute the energy spectrum.^[^ ^29^ ^]^ The topological phase diagram of a HOETI in Figure 2d is found to change smoothly as a function of the parameter ϕ ∈ [0, π/2] describing the couplings *w* and *v* via *w*/(*w* + *v*) = sin^2^(ϕ) and *v*/(*w* + *v*) = cos^2^(ϕ). For ϕ < π/4 (*w*/*v* > 1), the fractional end charge of a HOETI is proportional to the fractional corner charge as *Q*_end_ = 2*Q*_corner_ and two pairs of end‐localized energy‐degenerate in‐gap states are associated with the end charges. The global inversion symmetry protects the degeneracy of the end states and the additional local *C*_2_ symmetry pins the end states to gaps I and II, see Figure 2c. For ϕ > π/4 (*w*/*v* < 1), both the end charges and end states vanish. We thus have a trivial phase for *w*/*v* < 1 and a HOETI for *w*/*v* > 1. The end states originate from an anomalous spatial distribution of the nontrivial bulk states and result in an anomalous distribution of the charge density at 1/4 and 3/4 filling (Figure 2e), where an infinitesimal on‐site perturbation is introduced to split the degeneracy of the end states. The overall charge density is zero in the bulk and fractional end charges of ±1/2 are localized at the two ends of the RPL, representing the filling anomaly of a HOETI.

### 2.1 Origin from Topological Obstructions

**Figure 3:**
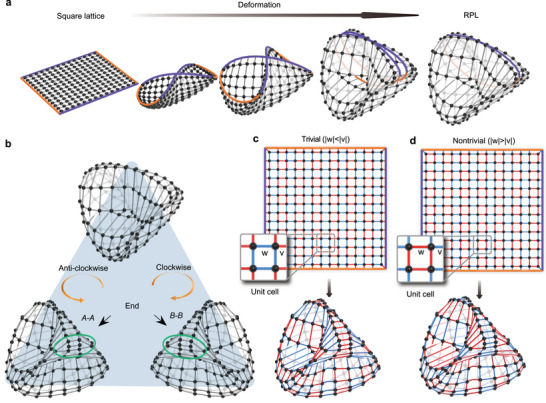
RPL. a) Deformation from a square lattice to the RPL. b) Rotations of the RPL with views of the *A*–*A* and *B*–*B* ends. c,d) Tight‐binding representations of the RPL with trivial and nontrivial unit cells consisting of four sites. The red and blue lines represent strong and weak couplings.

Mapping a model onto a topologically equivalent model allows a physical system to be pictured in a different, often more intuitive way. We visualize the RPL in a 3D Cartesian space and show that there are always end vertices corresponding to the end states in the RPL. **Figure** **3**a illustrates that a square lattice can be transformed into the RPL while maintaining the same real‐space topology. After rotating the RPL, the two ends form a pair of degenerate topological obstructions (merged diagonally opposite corners), as shown in Figure 3b. If *G* is a graph embedded in the RP^2^, its Euler characteristic is given by χ=#V−#E+#F, where #V, #E, and #F represent the numbers of vertices (sites), connections (nearest‐neighbor bonds), and faces (plaquettes) of the tiling, respectively. The RPL has a demigenus (or non‐orientable genus or Euler genus) of 1, independent of the embedding. One can determine the embedding of a quadrangular tessellation using the identities

(4) $$\begin{array}{ccc} \#V & = & \#V_{\mathrm{bulk}}+\#V_{\mathrm{end}}\\ 2\#E & = & 4\#V_{\mathrm{bulk}}+3\#V_{\mathrm{end}}\\ 4\#F & = & 4\#V_{\mathrm{bulk}}+3\#V_{\mathrm{end}} \end{array}$$

where the vertices consist of bulk vertices *V*_bulk_ and end vertices *V*_end_, with each connection linking two vertices and each face comprising four connections. Each *V*_bulk_ contributes to four adjacent faces and four connections, while each *V*_end_ contributes to three adjacent faces and three connections. Solving Equation (4), we find $\#V_{\mathrm{end}}=4$. Each of the four end vertices contributes equally to the topological invariant χ and corresponds to an end state in the RPL. The constraint $\#V_{\mathrm{end}}=4$ implies that the topological obstructions in the RPL are minimally represented by four end vertices, regardless of how the bulk vertices are arranged. However, the real‐space topology is only concerned with the existence of connections and not with their physical realization. In crystallography, graphs are used to describe the crystal structure, with the vertices and connections corresponding to atoms and bonds, respectively. As shown in Figure 3c (Figure 3d), both a trivial phase and a HOETI can be realized with the same 3D geometry but with different alternate couplings. The topological property of a HOETI is determined not only by the topological obstructions in the real space but also by the topological obstructions in the momentum space.^[^ ^30^ ^]^

### 2.2 Experimental Realization

**Figure 4:**
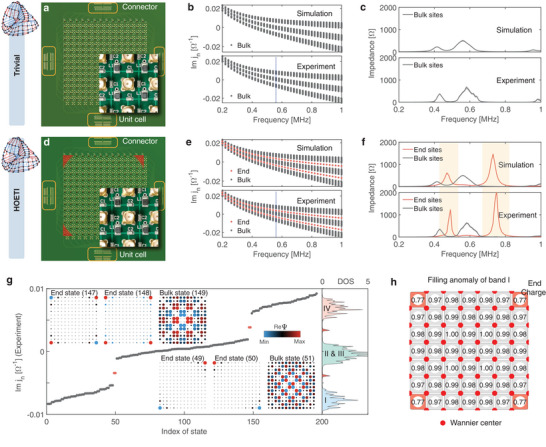
Experimental and simulated results. a,d) Topolectric circuit of the trivial phase (a) and HOETI (d). Insets show the unit cells. Note the different capacitors *C*_1_ and *C*_2_. Topological end states are expected to occur in the red areas. The boundary couplings are designed by connecting the connectors with DuPont lines. b,e) Admittance spectra obtained by simulation (top panels) and experiment (bottom panels) as functions of the driving frequency in the trivial phase (b) and HOETI (e). c,f) Impedance spectra obtained by simulation (top panels) and experiment (bottom panels) as functions of the driving frequency for the trivial phase (c) and HOETI (f). g) Experimental admittance spectrum and corresponding density of states (DOS) at the resonance frequency (blue dotted line in e). The insets show selected bulk and end states. h) Due to the filling anomaly, fractional end charges are observed for the HOETI. Analogous results for the trivial phase in Figure S5 (Supporting Information) show no filling anomaly, implying that there are no fractional end charges.

Since topolectric circuits are defined in terms of discrete elements and their connections,^[^ ^31^, ^32^, ^33^, ^34^ ^]^ a circuit can explicitly represent the RPL. We demonstrate the bulk‐end correspondence as a hallmark of a HOETI using coupling capacitors *C*_1_ = 1000 pF and *C*_2_ = 220 pF, and grounding inductors *L*_1_ = 33 µH. The circuits are shown in **Figures** **4**a,d, with the unit cells as the insets. The real projective boundary conditions are realized by connecting the connectors at the boundary with DuPont lines. Figures 4e (4b) and 4f (4c) show the admittance and impedance spectra obtained for the nontrivial (trivial) phase, respectively, demonstrating agreement between simulation and experiment. In the nontrivial phase, the red and grey dots represent the end and bulk states in the admittance spectra, respectively, and the red and grey curves represent the impedance spectra at the end and bulk sites. In the trivial phase, no evidence of end states or impedance peaks at the end sites is found. We obtain the admittance spectrum and corresponding density of states at the resonance frequency $f_{0}=1/2\pi \sqrt{2\left(C_{1}+C_{2}\right)L_{1}}$ with delocalized bulk states and localized end states (Figure 4g).

In addition to the localized end states, we demonstrate experimentally the fractional end charge as secondary evidence of the bulk‐end correspondence. Integrating the local density of states over the bulk bands and normalizing the result to the number of states in the unit cell yields a mode density analogous to charge density.^[^ ^35^, ^36^ ^]^ Considering band I, we find for each unit cell in the bulk region an integer charge, while in the end regions the charges are fractional (Figure 4h; see the Methods section for details). By adding the charges of diagonally opposite corners, we have

(5) $$Q_{\mathrm{end}}=2\rho_{\mathrm{corner}}\;\mathrm{mod}\;1$$

The experimental result of *Q*_end_ = 0.54 (≃ 1/2) confirms that the HOETI has a fractional end charge of 1/2.

## 3 Conclusion and Outlook

We have introduced the HOETI in the RPL as a new topological phenomenon. A HOETI is a dipole‐symmetry‐protected phase with a fractional end charge, extending the first‐order bulk‐end correspondence (0D end states with co‐dimension one) to higher order (0D end states with co‐dimension higher than one). Using topolectric circuits, we have observed a HOETI and confirmed the bulk‐end correspondence through the localized end states and fractional end charge. The discovery of a HOETI reveals the interplay between topological obstructions in real and momentum space, and lets the fractional end charge emerge as a powerful tool for probing higher‐order topology. Our design of topolectric circuits facilitates advanced Hamiltonian engineering, enables the realization of manifolds not accessible to the existing platforms, and paves the way to the emulation of uncharted physics in non‐Euclidean space.^[^ ^37^, ^38^, ^39^, ^40^, ^41^ ^]^

## 4 Methods

### Deformations and Fractional Charge

The design of ends through a cutting‐gluing procedure is illustrated in Figure S1 (Supporting Information). A square can be divided into four quadrants. By removing two quadrants and gluing the two remaining quadrants together, an oval‐shaped geometry can be obtained by deformation. In this way, a disclination with Frank angle Ω = −π can be created using two quadrants of a square lattice. In the RP^2^, two pairs of diagonally opposite quadrants can be deformed into two such geometries. Similarly, in the RPL, two pairs of corners of diagonally opposite quadrants can be deformed into two ends.

The deformation of the unit square ([0, 1] × [0, 1]) into the RP^2^ by twisting and gluing of the edges is shown in Figure S2 (Supporting Information). The 2D Cartesian coordinates can be mapped to latitude‐longitude coordinates as (*x*, *y*)↦(θ, φ) = (2π*x*, π(*y* − 1/2)) with θ ∈ [0, 2π] and φ ∈ [−π/2, π/2]. In 3D Cartesian coordinates, the RP^2^ can be denoted as

(6) $$\begin{array}{cc} & x\left(\theta ,\varphi \right)=\frac{1}{2}d^{2}\sin\left(2\theta \right)\sin^{2}\varphi \\ & y\left(\theta ,\varphi \right)=\frac{1}{2}d^{2}\sin\theta \cos\left(2\varphi \right)\\ & z\left(\theta ,\varphi \right)=\frac{1}{2}d^{2}\cos\theta \sin\left(2\varphi \right) \end{array}$$

where *d* is a constant. The implicit representation of the RP^2^ for *d* = 1 is^[^ ^42^ ^]^

(7) $$x^{2}y^{2}+x^{2}z^{2}+y^{2}z^{2}-xyz=0$$

The creation of fractional charges is illustrated in Figure S3 (Supporting Information). A tight‐binding model for a unit cell of four sites can describe a *C*_4_‐symmetric HOTI, which exhibits fractional charges of 1/2 at the edges and 1/4 at the corners. In the case of a HOETI, when the quadrants are combined with real projective boundary conditions, the fractional edge charges sum to identical integer charges everywhere in the bulk, while the fractional corner charges of 1/4 sum to a fractional end charge of 1/2.

In the trivial phases of the square lattice and RPL, see Figures S4a1–a3 and c1–c3 (Supporting Information), respectively, the eigenvalue spectrum shows no in‐gap states. As there is no filling anomaly, the Wannier centers do not give rise to fractional charges. In the HOTI, see Figure S4b1–b3 (Supporting Information), the eigenvalue spectrum shows in‐gap edge states and in‐band corner states. As there is a filling anomaly, the Wannier centers give rise to fractional edge and corner charges. The in‐band corner states are not robust due to hybridization with the bulk states. In the HOETI, see Figure S4d1–d3 (Supporting Information), the eigenvalue spectrum shows topologically protected in‐gap end states. As there is a filling anomaly, the Wannier centers give rise to fractional end charges.

### Topological Indices

In the presence of rotation symmetry the Bloch Hamiltonian *h*(**k**) satisfies $C_{n}h\left(k\right)C_{n}^{†}=h\left(R_{n}k\right)$, where *C*_*n*_ is the *n*‐fold rotation operator and *R*_*n*_ is an *n*‐fold rotation acting on the momentum **k**. At the high‐symmetry point Π^(*n*)^ satisfying *R*_*n*_Π^(*n*)^ = Π^(*n*)^ we have [*C*_*n*_, *h*(Π^(*n*)^)] = 0. Given the eigenstates *u*(Π^(*n*)^) of *h*(Π^(*n*)^), we therefore can calculate the eigenvalues of *C*_*n*_ at Π^(*n*)^ by diagonalizing the matrix 〈*u*_*l*_(Π^(*n*)^)|*C*_*n*_|*u*_*m*_(Π^(*n*)^)〉, where *l* and *m* run over the occupied bands. We denote these eigenvalues as $\Pi_{p}^{\left(n\right)}=e^{2\pi i(p-1)/n}$ (*p* = 1, …, *n*) and define the rotation invariants

(8) $$\left[\Pi_{p}^{\left(n\right)}\right]=\#\Pi_{p}^{\left(n\right)}-\#\Gamma_{p}^{\left(n\right)}$$

where $\#\Pi_{p}^{\left(n\right)}$ is the number of occupied bands with eigenvalue $\Pi_{p}^{\left(n\right)}$.

The crystalline topology can be deduced from the symmetry indicators (band representations).^[^ ^26^ ^]^ The primary topological index of a *C*_4_‐symmetric HOTI is^[^ ^12^ ^]^

(9) $$\chi^{\left(4\right)}=\left(\left[X_{1}^{\left(2\right)}\right],\left[M_{1}^{\left(4\right)}\right],\left[M_{2}^{\left(4\right)}\right]\right)$$

A disclination is characterized by the net translation (denoted by the Burgers vector *B*) and net rotation (denoted by the Frank angle Ω) accumulated under parallel transport of a vector along a loop enclosing the disclination. The secondary topological index of the disclination is given by^[^ ^43^ ^]^

(10) $$Q_{\mathrm{disclination}}=\frac{\Omega }{2\pi }\eta +B_{x}p_{y}-B_{y}p_{x}\;\mathrm{mod}\;1$$

where the polarizations *p*_*x*_ and *p*_*y*_ and the Wannier representation index η capture the topology of the occupied band. In the case of *C*_4_‐symmetry, we have^[^ ^13^ ^]^

(11) $$\eta =\left[X_{1}^{\left(2\right)}\right]+\frac{3}{2}\left[M_{3}^{\left(4\right)}\right]-\frac{1}{2}\left[M_{1}^{\left(4\right)}\right]$$

The topological invariant of a *C*_4_‐symmetric HOTI is governed by the following relations: i) The number of bands is constant, $\sum_{p}\#\Pi_{p}^{\left(n\right)}=\sum_{p}\#\Gamma_{p}^{\left(n\right)}$, i.e., $\sum_{p}\left[\Pi_{p}^{\left(n\right)}\right]=0$. ii) The time‐reversal symmetry implies $\left[M_{2}^{\left(4\right)}\right]=\left[M_{4}^{\left(4\right)}\right]$.^[^ ^12^ ^]^ With $p_{x}=p_{y}=\frac{1}{2}\left[X_{1}^{\left(2\right)}\right]\;\mathrm{mod}\;1$ and Ω = −π, the fractional end charge is

(12) $$Q_{\mathrm{end}}=\frac{1}{2}\left(-\left[X_{1}^{\left(2\right)}\right]+2\left[M_{1}^{\left(4\right)}\right]+3\left[M_{2}^{\left(4\right)}\right]\right)\;\mathrm{mod}\;1$$

The primary topological index is χ^(4)^ = (0, 0, 0) in the trivial phase and χ^(4)^ = (−1, 1, 0) in the nontrivial phase. Considering Equation (2), we find

(13) $$\begin{array}{c} Q_{\mathrm{end}}=2Q_{\mathrm{corner}} \end{array}$$

### Simulation and Experiment

The Agilent Design System software was employed for the numerical simulation of a circuit of 7 × 7 unit cells, using the exact values of the components in the fabricated sample. Chip multilayer ceramic capacitors of 1000 pF ±5% (Murata, GCM2165C1H102JA16D) and 220 pF ±5% (Murata, GCM2165C1H221JA16D) capacitance are chosen for realizing the alternate couplings. Wire‐wound inductors (Murata, LQH32NH330J23L) of 33 µH ±5% inductance, 1.14 Ω resistance, and a self‐resonance frequency above 20 MHz are chosen for the grounded inductors. The scattering matrix *S* of the circuit can be transformed into the circuit Laplacian $J^{-1}=Z_{0}\left(S+I\right){\left(I-S\right)}^{-1}$, where *Z*_0_ is the characteristic impedance and I is the identity matrix. In a scattering parameter measurement between two sites, the other sites are connected with 50 Ω load terminators to ensure zero reflection. The spectrum of *J* ia obtained by reconstructing *S* using a vector network analyzer (Tektronix TTr500) and physics‐graph‐informed machine learning.^[^ ^44^ ^]^

### Calculation of the Filling via the Local Density of States

To calculate the filling, we use the retarded Green's function $G_{R}\left(E\right)=\lim_{\varepsilon \to 0^{+}}{\left(E+i\varepsilon -H\right)}^{-1}$, where *H* is the Hamiltonian. *G*_*R*_(*E*) defines a meromorphic function of the parameter *E*.^[^ ^45^ ^]^ Defining *G*_*R*_(*r*, *r*′; *E*) = 〈*r*|*G*_*R*_(*E*)|*r*′〉, with *r* denoting the position, the local density of states is given by ρ_*r*_(*E*) = Im*G*_*R*_(*r*, *r*; *E*). After normalization to the number of states in the unit cell, we obtain the filling between *E*_*a*_ and *E*_*b*_ as

(14) $$\int_{E_{a}}^{E_{b}}\rho_{r}\left(E\right)dE/\int \rho_{r}\left(E\right)dE$$

In a circuit, the current *I*_*r*_ flowing into site *r* at frequency *f* follows Kirchhoff's law

(15) $$I_{r}=\sum_{r^{\prime }}J_{r,r^{\prime }}\left(2\pi f\right)V_{r^{\prime }}$$

where $V_{r^{\prime }}$ is the voltage at site *r*′. Considering that the ratio of the coupling capacitors equals the ratio of the coupling constants in the tight‐binding model, i.e., *C*_1_/*C*_2_ = *w*/*v*, we have^[^ ^46^ ^]^

(16) $$J\left(2\pi f_{0}\right)=i2\pi f_{0}C_{1}H$$

The filling is calculated using *H* = Im*J*(2π*f*_0_) and Equation (14).

## Conflict of Interest

The authors declare no conflict of interest.

## Author Contributions

C.S., S.L., C.J., and R.S. contributed equally to this work. C.S. conceived the idea and performed the theoretical analyses. C.S., S.L., and R.S. designed the circuits and performed the experiments. C.J. plotted the 3D figures. C.H.L., R.T., A.M., T.J.C., and U.S. guided the research. All the authors contributed to the discussions of the results and the preparation of the manuscript.

## Supporting information

Supporting Information

## Data Availability

The data that support the findings of this study are available from the corresponding author upon reasonable request.
