# Supplementary material for: Observation of a Higher‐Order End Topological Insulator in a Real Projective Lattice
Source: Adv Sci (Weinh). 2024 Jan 12;11(11):2303222. doi: 10.1002/advs.202303222 (PMC10953588; doi:10.1002/advs.202303222)
Supplement: Supplementary file 1 — Supporting Information [file ADVS-11-2303222-s001.pdf]

ADVANCED SCIENCE
Open Access

Supporting Information

for *Adv. Sci.*, DOI 10.1002/advs.202303222

Observation of a Higher-Order End Topological Insulator in a Real Projective Lattice

Ce Shang, Shuo Liu, Caigui Jiang, Ruiwen Shao, Xiaoning Zang, Ching Hua Lee, Ronny Thomale, Aurélien Manchon, Tie Jun Cui* and Udo Schwingenschlögl**

Supplementary Material: Observation of a higher-order end topological insulator in a real projective lattice

Ce Shang,[1,*] Shuo Liu,[2,*] Caigui Jiang,[3,*] Ruiwen Shao,[2,*] Xiaoning Zang,[1] Ching Hua Lee,[4,5,†] Ronny Thomale,[6,‡] Aurélien Manchon,[7,§] Tie Jun Cui,[2,¶] and Udo Schwingenschlögl[1,**]

[1]*King Abdullah University of Science and Technology (KAUST), Physical Science and Engineering Division (PSE), Thuwal 23955-6900, Saudi Arabia*

[2]*State Key Laboratory of Millimeter Waves, Southeast University, Nanjing 210096, China*

[3]*Institute of Artificial Intelligence and Robotics, Xi'an Jiaotong University, Xi'an 710049, China*

[4]*Department of Physics, National University of Singapore, Singapore 117551, Republic of Singapore*

[5]*Joint School of National University of Singapore and Tianjin University, International Campus of Tianjin University, Fuzhou 350207, China*

[6]*Institut für Theoretische Physik und Astrophysik, Universität Würzburg, Würzburg, Germany*

[7]*CINaM, Aix-Marseille University, CNRS, Marseille, France*

[*] These authors contributed equally

[†] phylch@nus.edu.sg

[‡] rthomale@physik.uni-wuerzburg.de

[§] aurelien.manchon@univ-amu.fr

[¶] tjcui@seu.edu.cn

[**] udo.schwingenschlogl@kaust.edu.sa

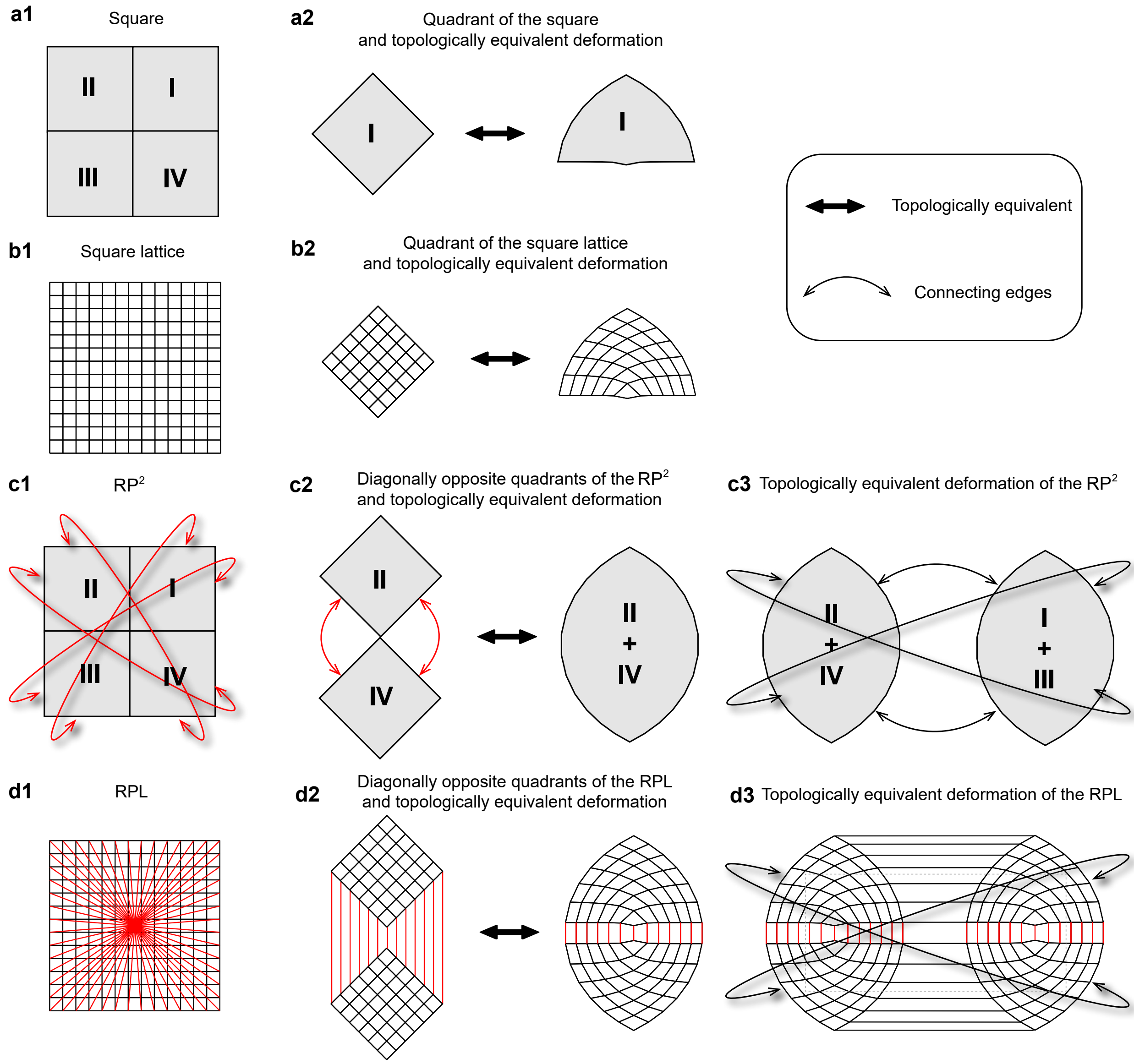

FIG. S1. Deformations. a1, Square with four quadrants. a2, Deformation of a quadrant into a topologically equivalent geometry. b1, Square lattice. b2, Deformation of a quadrant into a topologically equivalent lattice. c1, RP^2 with twisted gluing of opposite edges of a square. c2, Deformation of diagonally opposite quadrants with glued edges into a topologically equivalent disclination geometry. c3, Deformation of the RP^2 into two disclination geometries with twisted gluing. d1, RPL (square lattice with real projective boundary conditions). d2, Deformation of diagonally opposite quadrants with gluing rules into a topologically equivalent disclination lattice. d3, Deformation of the RPL into two disclination lattices with twisted gluing. The grey dashed rectangle marks the area shown in Fig. 1d. The gluing rules are marked in red. Crossing arrows mean twisted gluing.

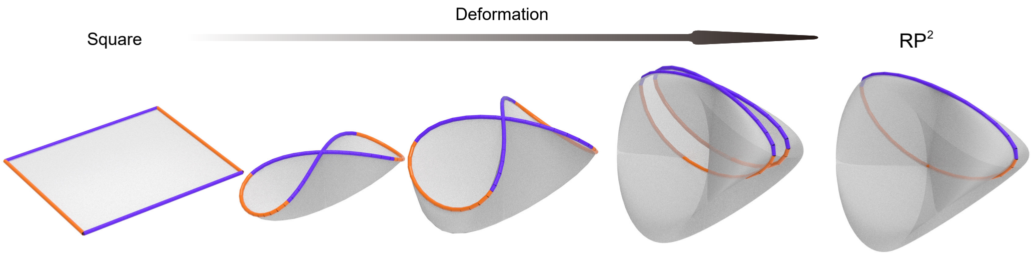

FIG. S2. Deformation from a square to the RP^2.

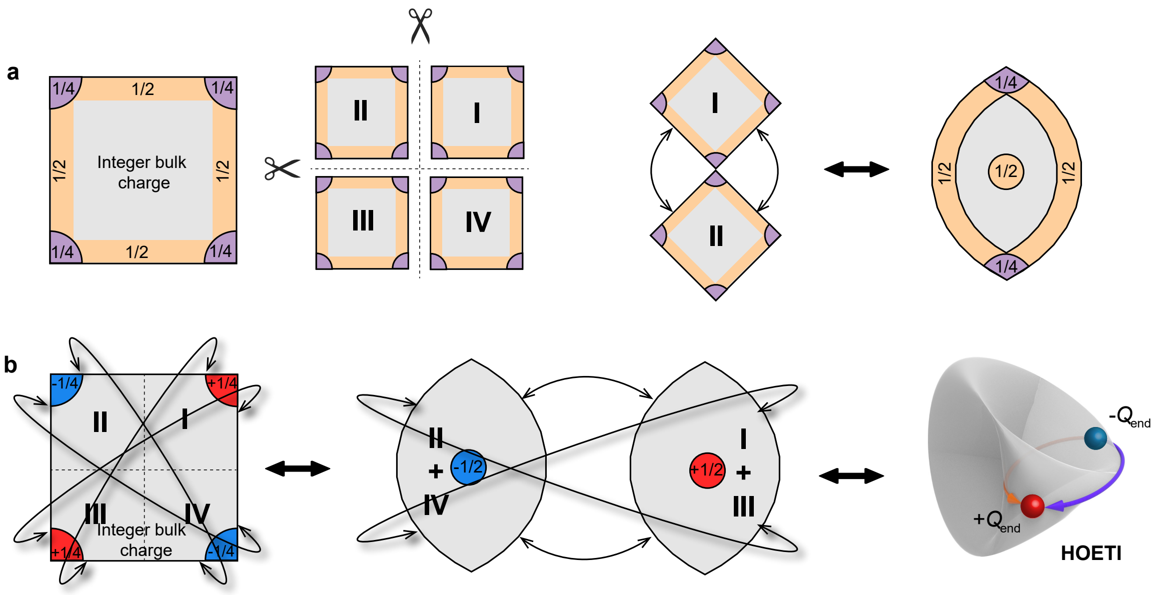

FIG. S3. Fractional charge. a, C_4-symmetric HOTI with corner charges of 1/4 and edge charges of 1/2. After cutting into four identical quadrants, two quadrants are removed and the remaining quadrants are glued together to create a disclination. When the lowest band is filled, the disclination traps a fractional charge of 1/2, as it is formed by combining two corner charges of 1/4. b, HOETI with end charges of ±1/2. At 1/4 filling, the ends trap fractional charges of ±1/2, as they are formed by combining two pairs of corner charges.

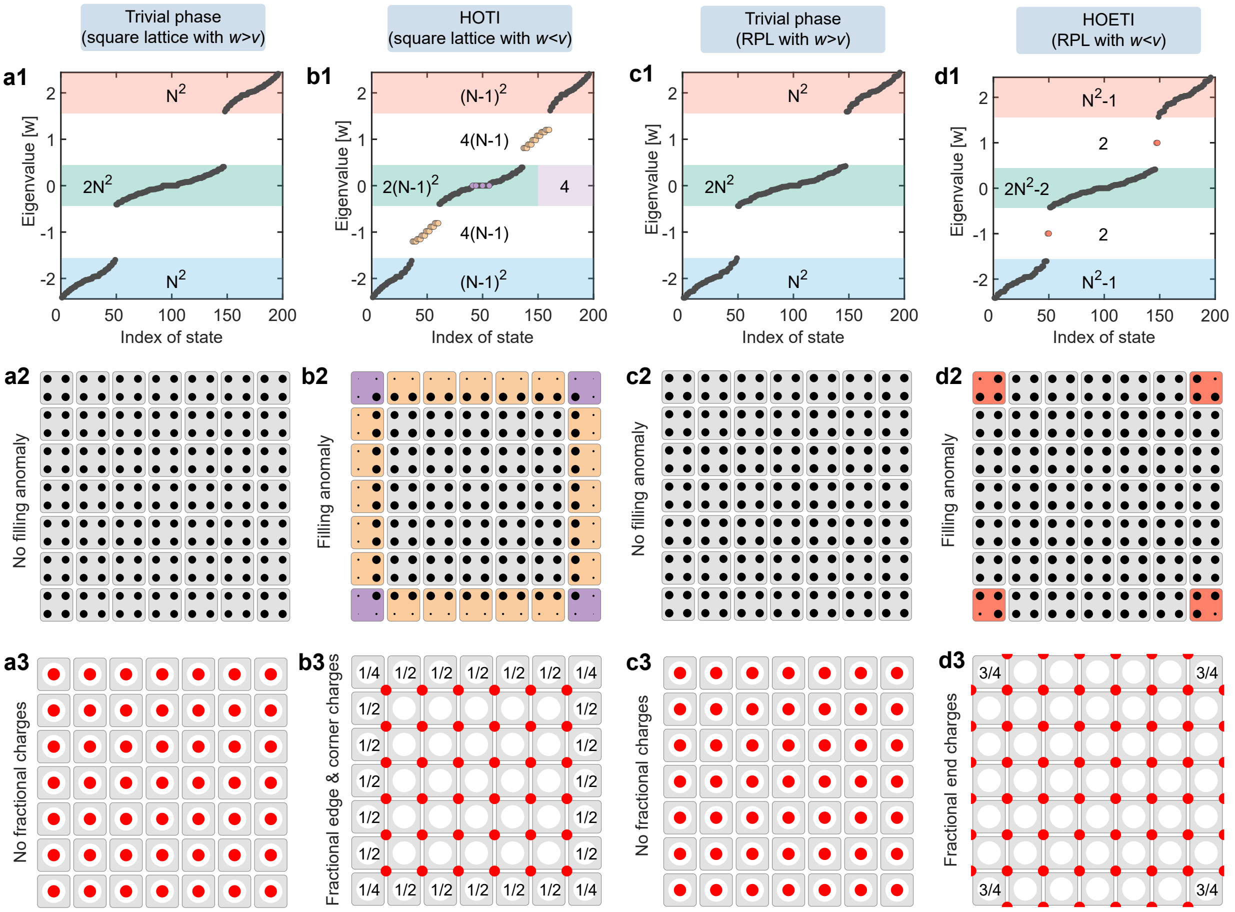

FIG. S4. Comparison of HOTI and HOETI. a1-a3, c1-c3, The eigenvalue spectrum shows no in-gap states. Due to the fact that there is no filling anomaly, the Wannier centers (red circles) do not result in fractional charges. b1-b3, The eigenvalue spectrum shows in-gap edge states and in-band corner states. Due to the filling anomaly, the Wannier centers result in fractional edge and corner charges. d1-d3, The eigenvalue spectrum shows only in-gap end states. Due to the filling anomaly, the Wannier centers result in fractional end charges.

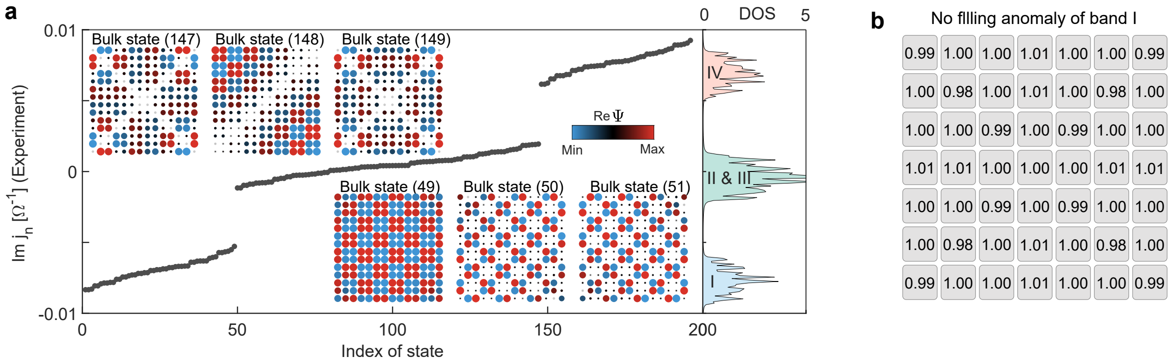

FIG. S5. Trivial phase. a, Experimental admittance spectrum and corresponding density of states (DOS) at the resonance frequency (blue dotted line in Fig. 4b). The insets show selected bulk states. b, Due to the fact that there is no filling anomaly, no fractional charges are observed.
